# Supplementary material for: Application of QUBO solver using black-box optimization to structural design for resonance avoidance
Source: Sci Rep. 2022 Jul 15;12:12143. doi: 10.1038/s41598-022-16149-8 (PMC9287372; doi:10.1038/s41598-022-16149-8)
Supplement: Supplementary file 1 — Supplementary Figures. [file 41598_2022_16149_MOESM1_ESM.pdf]

Supplementary Information
Application of QUBO solver using black-box optimization to structural design for resonance avoidance

Tadayoshi Matsumori[1,*], Masato Taki[1], and Tadashi Kadowaki[1]

[1]DENSO CORPORATION, 500-1, Minamiyama, Komenoki-cho, Nisshin, Aichi, 470-0111, Japan

*tadayoshi.matsumori.j7b@jp.denso.com

This supplementary information provides all data which we used for drawing Figs 4, 7a, 8b, 9, and 10 in the main text. In this study, we demonstrated the performance of the black-box optimization for a QUBO solver through the design problems for two simplified printed circuit board models with 17 and 27 mounting holes. The design problems were formulated in the multi-objective optimization methods, i.e., the weighted sum method and the ε-constraint method. And they were reformulated into QUBO using the black-box optimization methods, the factorization machine (FM) and Bayesian optimization of combinatorial structures (BOCS), which were called FM-QUBO and BOCS-QUBO, respectively. Each design problem was solved 10 or 20 times with different initial data set using FM-QUBO, BOCS-QUBO, and random search, and their optimized results were shown in Figs 4, 7a, and 8b in "Results". In addition, we solved the design problem with 17 mounting holes using simulated annealing and quantum annealing and changing the value of the hyper-parameter in FM to confirm the effect of the QUBO solver and the hyper-parameter in FM on the performance of FM-QUBO. Figures. 9, and 10 in "Discussion" showed their optimized results. Figures 4, 7a, 8b, 9, and 10 were illustrated the average objective function values of the best solution and their 95% confidence interval.

Figures S1 to S7 show all the optimization histories before the statistical analysis which we used for drawing Figs. 4, 7a, 8b, 9, and 10. In each of the figures, "No." shown in the figure legends represents the identification number when the optimization was conducted 10 or 20 times with different initial values. The same "No." in each of the figures means that an optimization started from the same initial data set.

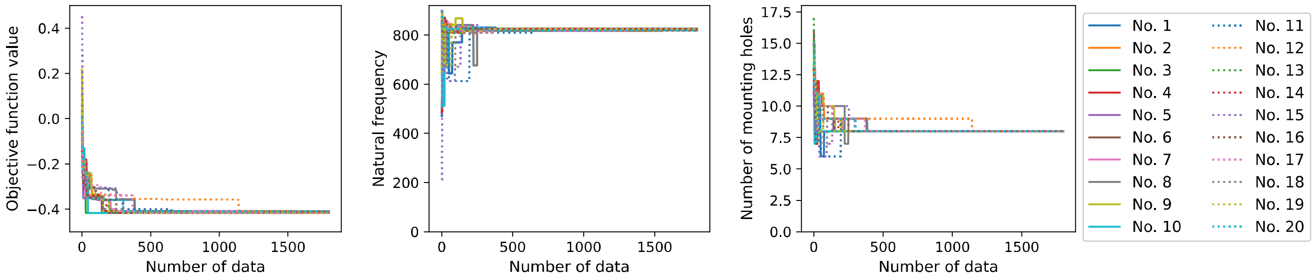

(a) FM-QUBO

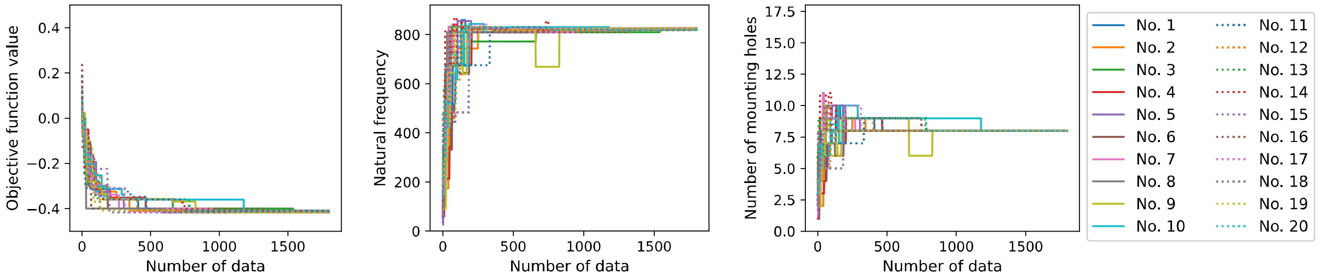

(b) BOCS-QUBO

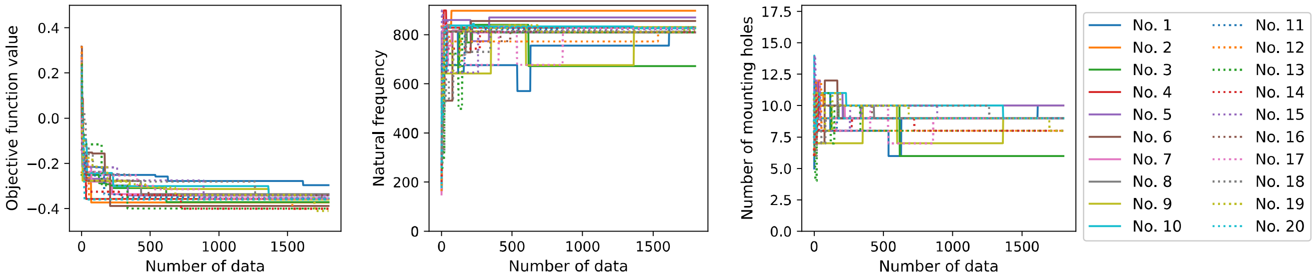

(c) Random search

Figure S1 The optimization histories of the QUBO formulated by the weighted sum method with $n = 17$ design variables and $w = 0.5$. The average objective function values of the best solution and their 95% confidence interval are shown in Figure 4 in the main text.

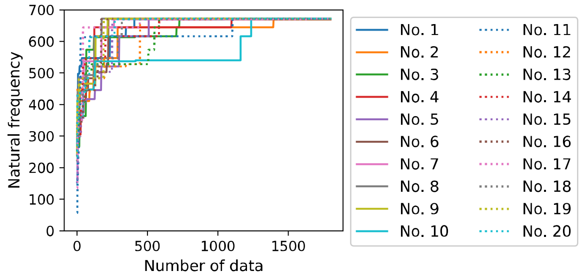

(a) FM-QUBO

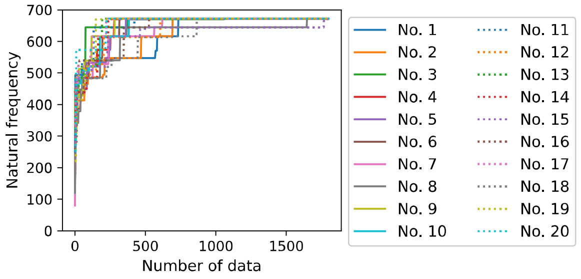

(b) BOCS-QUBO

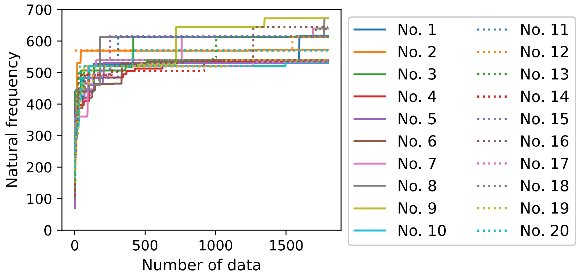

(c) Random search

Figure S2 The optimization history of the QUBO formulated by the ε-constraint method with $n = 17$ design variables and $\overline{N} = 6$. The average objective function values and their 95% confidence interval are shown in Figure 7a in the main text.

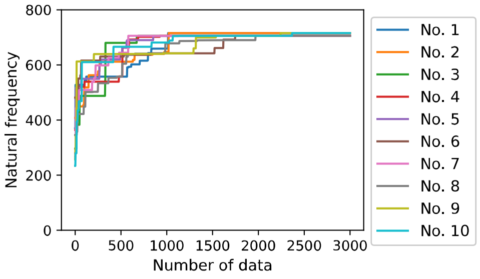

(a) FM-QUBO

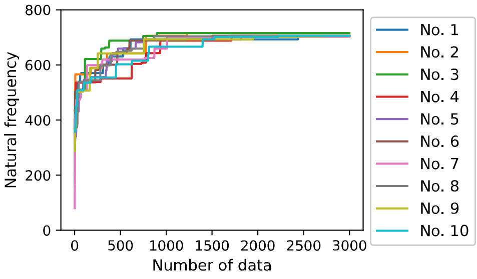

(b) BOCS-QUBO

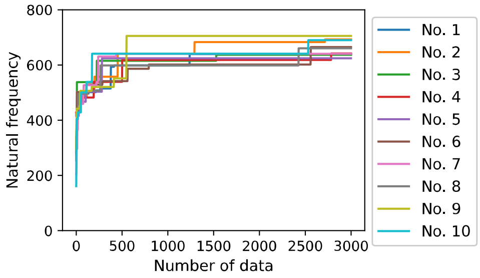

(c) Random search

Figure S3 The optimization history of the QUBO formulated by the ε-constraint method with $n = 27$ design variables and $\overline{N} = 8$. The average objective function values and their 95% confidence interval are shown in Figure 8b.

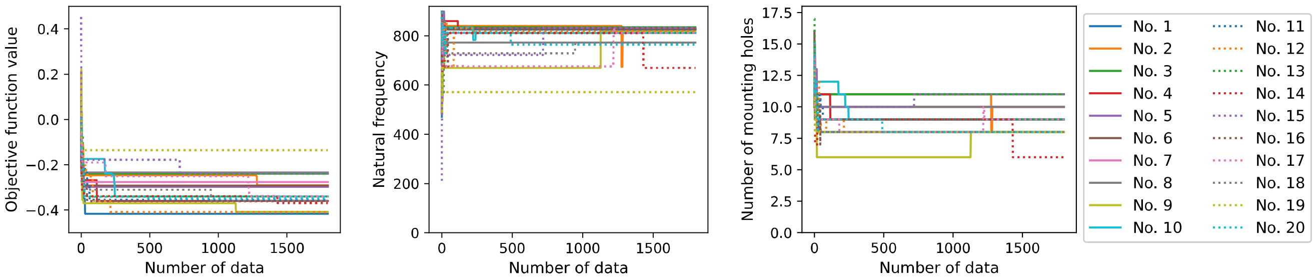

(a) Simulated annealing (the optimized solution was only added.)

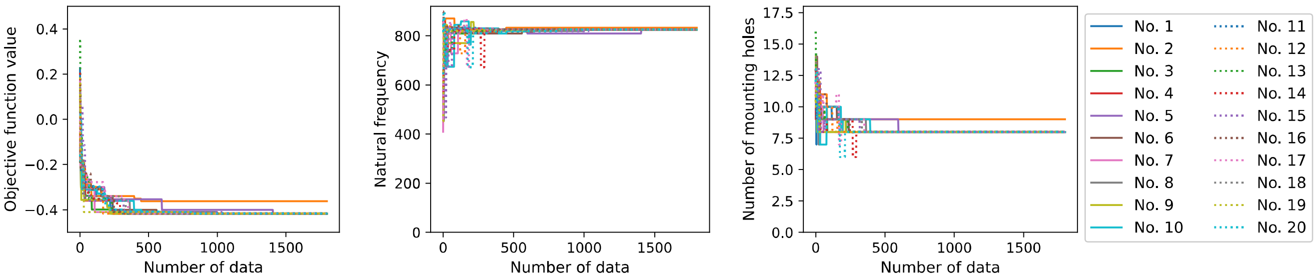

(b) Quantum annealing

Figure S4 The optimized results of the FM-QUBO using simulated annealing and quantum annealing. When FM was updated during the optimization, (a) in the simulated annealing, the optimized solution was only added, while (b) in the quantum annealing, the optimized solution and its two neighbors were added. The QUBO was formulated by the weighted sum method with $n = 17$ design variables and $w = 0.5$. The average objective function values and their 95% confidence interval are shown in Figure 9a in the main text.

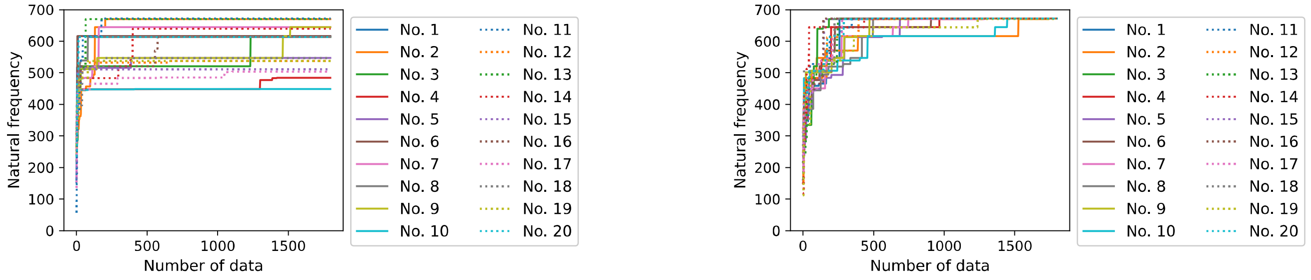

(a) Simulated annealing
(the optimized solution was only added.)

(b) Quantum annealing

Figure S5 The optimized results of the FM-QUBO using simulated annealing and quantum annealing. The QUBO was formulated by the ε-constraint method with $n = 17$ design variables and $\overline{N} = 6$. When FM was updated during the optimization, (a) in the simulated annealing, the optimized solution was only added, while (b) in the quantum annealing, the optimized solution and its two neighbors were added. The average objective function values and their 95% confidence interval are shown in Figure 9b.

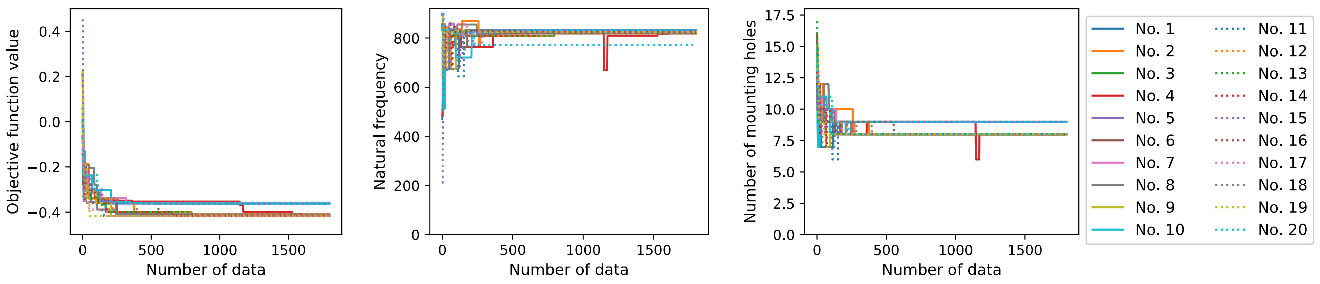

(a) $k = 9$

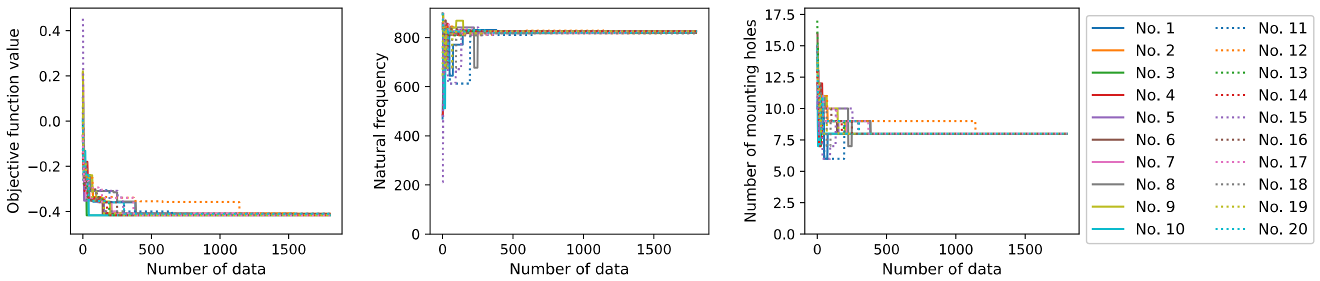

(b) $k = 12$

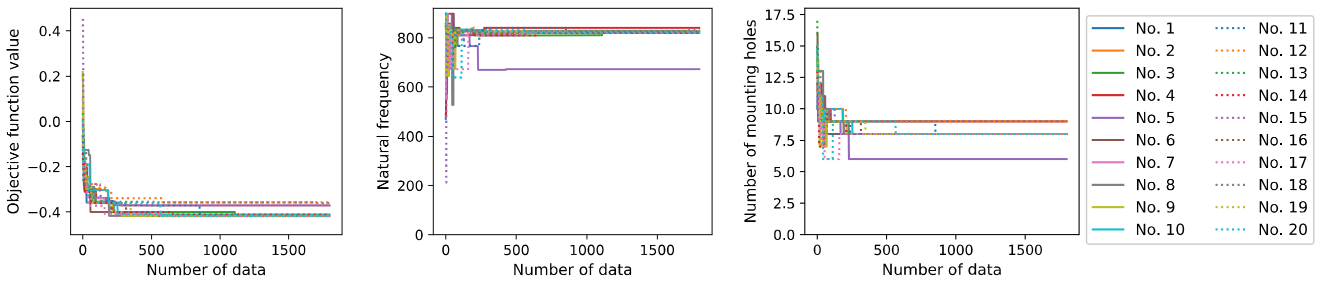

(c) $k = 15$

Figure S6 The optimized results of the FM-QUBO with $k = 9, 12, 15$. The QUBO was formulated by the weighted sum method with $n = 17$ design variables and $w = 0.5$. The average objective function values and their 95% confidence interval are shown in Figure 10a in the main text.

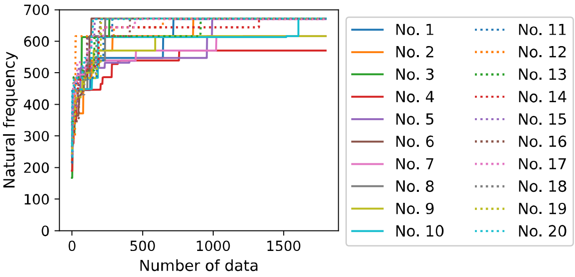

(a) $k = 9$

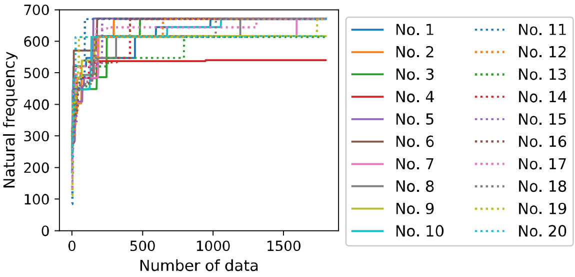

(b) $k = 12$

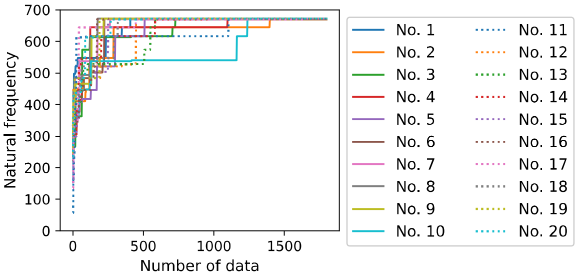

(c) $k = 15$

Figure S7 The optimized results of the FM-QUBO with $k = 9, 12, 15$. The QUBO was formulated by the ε-constraint method with $n = 17$ design variables and $\overline{N} = 6$. The average objective function values and their 95% confidence interval are shown in Figure 10b.
